# Robust SAR Automatic Target Recognition Based on Transferred MS-CNN with L^2^-Regularization

**DOI:** 10.1155/2019/9140167

**Published:** 2019-11-15

**Authors:** Yikui Zhai, Wenbo Deng, Ying Xu, Qirui Ke, Junying Gan, Bing Sun, Junying Zeng, Vincenzo Piuri

**Affiliations:** ^1^Department of Intelligent Manufacturing, Wuyi University, Jiangmen 529020, China; ^2^School of Electronics and Information Engineering, Beihang University, Beijing 100191, China; ^3^Dipartimento di Informatica, Universita' Degli Studi di Milano, Via Celoria 18, 20133 Milan, Italy

## Abstract

Though Synthetic Aperture Radar (SAR) Automatic Target Recognition (ATR) via Convolutional Neural Networks (CNNs) has made huge progress toward deep learning, some key issues still remain unsolved due to the lack of sufficient samples and robust model. In this paper, we proposed an efficient transferred Max-Slice CNN (MS-CNN) with L^2^-Regularization for SAR ATR, which could enrich the features and recognize the targets with superior performance. Firstly, the data amplification method is presented to reduce the computational time and enrich the raw features of SAR targets. Secondly, the proposed MS-CNN framework with L^2^-Regularization is trained to extract robust features, in which the L^2^-Regularization is incorporated to avoid the overfitting phenomenon and further optimizing our proposed model. Thirdly, transfer learning is introduced to enhance the feature representation and discrimination, which could boost the performance and robustness of the proposed model on small samples. Finally, various activation functions and dropout strategies are evaluated for further improving recognition performance. Extensive experiments demonstrated that our proposed method could not only outperform other state-of-the-art methods on the public and extended MSTAR dataset but also obtain good performance on the random small datasets.

## 1. Introduction

SAR ATR is widely used in various fields such as urban monitoring, natural environment survey, and military target reconnaissance [1, 2], which can acquire earth observation images from severely adverse weather conditions and excavate hidden and camouflaged targets effectively [3, 4]. Compared with other microwave detection tools, distinctive characteristics derived from SAR images can work well better than other sensors, like optical and infrared methods on coherent imaging system and electromagnetic scattering mechanism. Nowadays, SAR ATR is an essential technique in remote sensing application.

SAR ATR was restricted by imaging quality and advancement of image classification. SAR target classification can be categorized as traditional methods and deep learning methods. Generally, traditional methods aim to extract discriminative and represented features from the training samples. Traditional feature extraction methods, such as Histogram of Oriented Gradients (HOG) [5], Local Binary Pattern (LBP) [6], Principal Component Analysis (PCA) [7], and Scale Invariant Feature Transform (SIFT) [8], were applied to SAR target classification task. Song et al. [9] designed a novel gradient HOG-like feature-based SAR ATR method to tackle a complex application environment. Li et al. [10] proposed a HOG descriptor-based method to match features between SAR images and optical images. Ghannadi and Saadatseresht [6] proposed a modified LBP descriptor to obtain robust features for SAR ATR. Wang et al. [11] presented an improved SAR interferogram denoising method based on PCA to improve the accuracy of phase unwrapping. Xiang et al. [12] combined an adaptive sampling method with SAR-SIFT to eliminate obvious scale difference images. However, the presence of speckle noises and lack of robust features have seriously degraded the feature robustness of the SAR image.

In recent years, deep learning methods have been proposed to extract more robust features. Rumelhart et al. [13] proposed the Back Propagation (Back Propagation) algorithm for Multilayer Perception (Multilayer Perception, MLP) to effectively solve the nonlinearity classification problem. LeCun et al. [14] presented LeNet structure to improve the classification performance. In 2006, Hinton et al. [15] proposed a self-learning method to overcome the gradient disappearance in deep network training. Zhang et al. [16] proposed a SAR-CNN model with batch normalization to estimate the speckled image and improve its performance. Zhen et al. [17] integrated effective image preprocessing and CNN for SAR target classification. Shahzad et al. [18] used CNN to generate visible images with high quality from SAR images and yield good result. Hughes et al. [19] identified corresponding patches in SAR and optical images with pseudosiamese CNN, to identify corresponding patches in high-resolution optical and SAR remote sensing imagery. Though the above methods could achieve relatively good performance, two key problems still remain unsolved in SAR ATR [17, 20].

The first challenging problem is effective model design, which is mainly impacted by objective function and cost function design, super-high dimensional parameters optimization, and so on. The second challenging problem is model generalization, such as overfitting caused by insufficient samples and generalization ability on unknown targets.

In this paper, a transferred MS-CNN with L^2^-Regularization is proposed to tackle the aforementioned challenging problems. Firstly, joint ROI feature extraction and data amplification methods are adopted to prepossess interested SAR regions and enhance the richness of raw training samples, respectively, which could seek accurate interested image regions and effectively scale up the dataset. Secondly, a novel transferred MS-CNN framework with L^2^-Regularization is proposed to extract robust features and address the overfitting challenge. Thirdly, transfer learning is employed to improve the feature discrimination and evaluate the model robustness. Then, dropout strategy is utilized to address the redundancy of extracted features from the network. Finally, experiments conducted on the original and extended MSTAR dataset indicated that the proposed method could achieve an excellent performance. The contributions of this paper can be summarized as follows:

1. Joint ROI and data amplification: ROI extraction and data amplification methods are presented to suppress noise and enrich numbers of raw training samples
2. Max-Slice CNN model: the presented MS-CNN model could not only extract robust features but also recognize the targets correctly on MSTAR database and yield satisfactory performance on both small samples
3. L^2^-regularization: L^2^-regularization algorithm is incorporated to avoid overfitting and optimize the trained model, which could boost 8.53% compared with the one without L^1^-regularization
4. Transfer learning strategy: transfer learning is employed for improving the robustness under small samples and outperforms other state-of-the-art methods, thus greatly increasing the performance of the feature generalization representation and discrimination

The rest of the paper is mainly organized as follows: Section 2 introduces the related work on SAR ATR.  Section 3 describes the proposed method in SAR ATR.  Section 4 presents the content of transfer learning.  Section 5 details the conducted experiments.  Section 6 draws the conclusions.

## 2. Related Work

### 2.1. Convolutional Neural Networks

Convolution neural network is a forward neural network [21] through convolution operation to realize the connection between network layers [22], which incorporates Convolutional layers (Convs), Rectified Linear Unit (Rectified Linear Unit, ReLU) layers, Pooling layers (Pooling), Fully Connection layers (FC), and so on. The Convs applied linear filters followed by activation functions, such as Randomized Parameterized ReLU, Exponential Linear Units (ELU), Scaled Exponential Linear Units (SELU), TanHyperbolic (Tanh), and so on. The filter weights were shared across receptive fields in the Convolutions. The activation layer was adopted to increase nonlinearity of the network without affecting receptive fields of Conv layers. The pooling layer was applied to nonlinear activation maps, and useless information or redundancy in feature maps was discarded. The fully connection layer maps extracted visual features to desired outputs and generated a value to represent grasp success probability. Thus, CNN could extract low-level features from images from early layers, which provided justification behind development of other improved CNNs.

GoogleNet has achieved a significant recognition effect on large-scale visual recognition [23] by using maximum and average pooling, random inactivation, and softmax classifier. A residual module [24] was proposed to enhance feature learning by jumping connection and prevent the gradient dissipation. The inception module [22] extracted multiscale information from an image by convolution operation of different branches to widen the network. In addition, the inception-v4 [25] structure formed by introducing jump connection into inception could greatly accelerate training speed and improve network performance. The pyramid model [26], which was composed of bottom-up, top-down repetitive processing and intermediate supervision, was also proposed to improve the performance by processing and integrating features on a multiscale architecture. Liu et al. [27] analyzed the performance of GoogleNet in SAR ATR and achieved good results. Wang et al. [28] used very deep convolutional networks (VGG) to construct ship classification in SAR images to solve training bottleneck caused by small dataset. Fu et al. [29] used learning optimization in ResNet for SAR ATR to solve the feature extraction challenge. CNNs rely on training samples, while insufficient training data result in an overfitting phenomenon.

### 2.2. Regularization

Due to overfitting caused by insufficient samples and large scale of parameters, regularization methods have become a significant strategy in deep learning to improve model generalization. Regularization technique, which discourages complexity of the model, can be categorized as L^1^ and L^2^-regularization. Their difference lies in the parameter restricted term. L^1^-regularization, also referred to as L^1^ norm or Lasso, helps shrink the parameters to zero and finish feature selection by assigning insignificant input features with zero weight and useful features with a nonzero weight. L^2^-regularization is the sum of square of all feature weights to fix the error by penalizing the weights. Regularization is a good method less prone to overfitting and wildly used for deep learning models.

Bi et al. [30] used the L^1^-regularization-based SAR imaging and CFAR detection method to efficiently improve SAR imaging performance, including suppressing sidelobes and clutter. Rambour et al. [31] introduced spatial regularization in SAR tomography reconstruction to sever to the ground analysis. Wagner et al. [32] proposed a deep learning SAR ATR system using regularization and prioritized classes to improve the convergence properties. Meng et al. [33] adopted an adaptive pseudo-p-norm regularization based despeckling SAR images method to provide a high-quality interpretation of SAR data. Ni et al. [34] presented the L^1^/L^2^-regularization SAR imaging via complex image data to better reconstructing the image target detection task. Kang and Kim [35] used improved L^2^-regularization for compressive sensing to enhance the performance. Although regularization is a good choice to avoid model overfitting in SAR ATR and recognition machine, feature extraction and feature discrimination methods are still challenging issues accurately distinguishing targets.

### 2.3. Transfer Learning

Transfer learning [36] is an important research topic in machine learning. The goal of transfer learning is to transform the knowledge learned from a domain to different but related ones and to reuse the knowledge of target domain by using shared information from source domain. The transfer learning method can effectively use existing marked data to assist classification task of similar datasets and improve target recognition rate of SAR images. Therefore, it can effectively alleviate the intervention of inherent factors and shortage of tagged training samples, and provide an effective path to improve target recognition performance.

Wang et al. [37] adopted transfer learning for SAR target detection based on SSD with data augmentation and obtained better performance than other methods. Zhong et al. [38] presented a simple and feasible approach by using transfer learning and achieved a good performance. Xu et al. [39] proposed a differentiated adaptive regularized transfer learning framework for SAR ship classification to overcome the limitation under insufficient labeled training samples. Al Mufti et al. [40] employed a pretrained AlexNet to train a multiclass SVM classifier. Thus, transfer learning is becoming a popular approach to solve small sample problems.

## 3. Proposed Method

**Figure 1:**
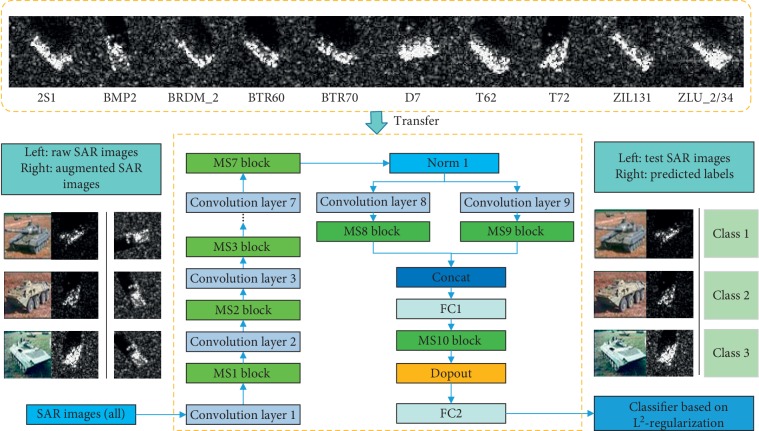
Structure of transferred MS-CNN for SAR target recognition. Firstly, raw SAR images and transformed SAR images is utilized in MS-CNN to extract information, and the extracted features are by normalization operation to preprocess the learned features. Then, the learned features are transformed to Max-Slice block; and the obtained feature maps are scaled to different size and operated with feature aggregation; meanwhile, the processed feature maps are merged and associated with each specified size. Thirdly, various filters are utilized to obtain the feature information and max-pooling is served to enforce the robustness of the features. The fully connected high-level feature layer and softmax layer predict the recognized classes. Finally, parameters from outside datasets are transferred to the target classification.

In our work, the transferred MS-CNN method is proposed to develop a feature refinement from the initial SAR image to the final classification map. The structure incorporates training stage and testing stage. During the training stage, the input of the transferred MS-CNN is the enriched samples augmented by ROI and data amplification and the output is the corresponding predicted label; a softmax classifier with L^2^-regularization is considered as the loss function to optimize the network. During the testing stage, images and labels are input into the network, and the aim is to extract the features by using the learned model and predict the recognized classes. The framework is shown in Figure 1.

The operation of convolution in MS-CNN is shown as

(1) $$\begin{array}{c} y^{s}=\max\left(0,\sum_{k}x^{k}\;⊗\;w^{k,s}+b^{s}\right), \end{array}$$

where *x* ^*k*^ and *y* ^*s*^ indicate that feature maps are extracted from the *k-*th input and *s*-th feature maps, respectively. *W* is described as the convolutional filter connecting the *k*-th input feature map and *s*-th output map. ⊗ Denotes the operation of convolution. *b* ^*s*^ is the bias of the *j*-th output map. For learning various regional features, weights in each layer are locally shared. In addition, max-pooling is illustrated as

(2) $$\begin{array}{c} y_{i,j}^{s}=\underset{0\leq m,n\leq p}{\max}x_{i\cdot p+m,j\cdot p+n}^{s}, \end{array}$$

where each value in the *s*-th output map *y* ^*s*^ pools over the *p* × *pn*-overlapping region in the *s*-th input map *x* ^*s*^.

In the training stage, images input into the network are processed layer wise to obtain the representative features, to have the data further intuitionistic. As is shown in Table 1, data visualization is employed by using this method.

### 3.1. Data Preprocess and Amplification

Suffering from the background noise, especially speckle noise performs negative to the classification task. ROI extraction from the SAR targets, via resizing the input samples, contributes to reducing the influence of irrelevant background noise and optimizing the training time and coverage speed. The ROI in SAR image stands in the central region in the whole picture, and then the ROI algorithm is employed below to obtain the interested region. The particle of the image is considered as the center to locate the target. The formula is shown in the following equation:

(3) $$\begin{array}{c} \left(i_{c},j_{c}\right)=\left(\frac{m_{10}}{m_{00}},\frac{m_{01}}{m_{00}}\right), \end{array}$$

where (*i*_*c*_, *j*_*c*_) is the particle ordinations of SAR target image. The *m*_10_ and *m*_01_ are 1-order origin moments.

(4) $$\begin{array}{c} m_{ij}=\underset{x}{\sum }\sum_{y}x^{i}x^{j}p\left(x,y\right), \end{array}$$

where *m*_*ij*_ denotes the (*i* + *j*)-order origin moment, (*x*, *y*) denotes the pixel coordinates of the image, and *p* (*x*, *y*) denotes the pixel value.

**Figure 2:**
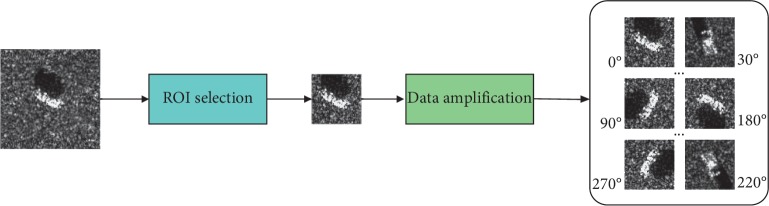
The procedures of data process and amplification.

To the best of our knowledge, feature extraction and classifier discrimination are also affected by limitation of training sample. The adequacy and confidence of feature information determine the classification performance as well. To this regard, a data amplification method that could rotate the target image at 360 degrees is proposed to address the challenges in sufficient training samples. This method could generate superior SAR target images that can not only better remove background noise from images and locate the region interested to process but also can offset the disadvantages caused by insufficient samples and avoid overfitting as well as gradient explosion. The data process and amplification procedure are depicted in Figure 2.

### 3.2. MS Block for Feature Refinement

The MS-block is used to refine the extracted features from convolution layers. The role of the slice layer is to decompose the bottom into multiple tops as needed. Considering that the dimensions of the input feature are the *N* *∗* 2 *∗* *H* *∗* *W*, set the axis to be the value 1, that is, the dimension to be decomposed of the feature map. Under the basis of slice operation, the dimensions of the output feature map will be *N* *∗* 1 *∗* *H* *∗* *W* and *N* *∗* 1 *∗* *H* *∗* *W*, respectively. The aim of the designed eltwise layer is to obtain the refined feature maps with the same dimensions as the output of convolution layers by element operation. Three basic operations SUM, PROD, and MAX are available to make its realization. In this paper, MAX operation served to compare the size of output feature maps for the goal of obtaining refined feature maps.

**Figure 3:**
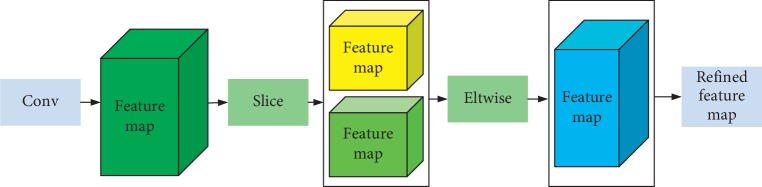
Structure of the MS-block.

The achievements of MS-block are described in Figure 3. Firstly, the scale space of the MS-block representation is obtained by smooth convolution with different Gaussian kernels and shares the same resolution on all scales. Secondly, due to the redundant information produced by the block, max pooling is provided to reduce the redundancy and increase the efficiency. Thirdly, the advantage of feature representation is that the local features of the image can be described on different scales in a simple form with an abundant theoretical basis to analyze the local features of the image.

### 3.3. L^2^-Regularization-Based Classifier

After the MS-CNN is amply trained, an L^2^-regularization-based classifier is employed to recognize the SAR images. Consider that *N* input variables are represented by vector *V*. The prediction can be illustrated as follows:

(5) $$\begin{array}{c} P\left(Y=y\;\left|\;v\right.\right)=\text{soft}\max{\left(W^{T}v+b\right)}_{y}, \end{array}$$

where *W* is the weights of above layers and *T* is transposition operation. *b* is the bias of the output map.

Assuming that the index, a class of submodels based on the element-by-element penalty of a binary vector, is *d*, the formula can be described as follows:

(6) $$\begin{array}{c} P\left(Y=y\;\left|\;v\right.;d\right)=\text{soft}\max{\left(W^{T}\left(d\;⊙\;v\right)+b\right)}_{y}. \end{array}$$

The integrated predictor is defined as the geometric average of the predictions of all members restandardized as follows:

(7) $$\begin{array}{c} P_{\text{ensemble}}\left(Y=y\;\left|\;v\right.\right)=\frac{{\tilde{P}}_{\text{ensemble}}\left(Y=y\;\left|\;v\right.\right)}{\sum_{y^{\prime }}{\tilde{P}}_{\text{ensemble}}\left(Y=y^{\prime }\;\left|\;v\right.\right)}, \end{array}$$

where

(8) $$\begin{array}{c} {\tilde{P}}_{\text{ensemble}}\left(Y=y\;\left|\;v\right.\right)=\sqrt[{2^{n}}]{\underset{d\in {\left\{0,1\right\}}^{n}}{\prod }P\left(Y=y\;\left|\;v\right.;d\right)}. \end{array}$$

Formula (10) is simplified as follows:

(9) $$\begin{array}{c} {\tilde{p}}_{\text{ensemble}}\left(Y=y\;\left|\;v\right.\right)=\sqrt[{2^{n}}]{\underset{d\in \left\{0,1\right\}}{\prod }P\left(Y=y\;\left|\;v\right.;d\right)},\\ =\sqrt[{2^{n}}]{\underset{d\in {\left\{0,1\right\}}^{n}}{\prod }\text{soft}\max{\left(W^{T}\left(d\;⊙\;v\right)+b\right)}_{y},}\\ =\sqrt[{2^{n}}]{\underset{d\in \left\{0,1\right\}}{\prod }\frac{\exp\left(W_{y,:}^{T}\left(d\;⊙\;v\right)+b\right)}{\sum_{y^{\prime }}\exp\left(W_{y\prime ,:}^{T}\left(d\;⊙\;v\right)+b\right)}},\\ =\frac{\sqrt[{2^{n}}]{\prod_{d\in {\left\{0,1\right\}}^{n}}\exp\left(W_{y,:}^{T}\left(d\;⊙\;v\right)+b\right)}}{\sqrt[{2^{n}}]{\prod_{d\in {\left\{0,1\right\}}^{n}}\sum_{y^{\prime }}\exp\left(W_{y\prime ,:}^{T}\left(d\;⊙\;v\right)+b\right)}}. \end{array}$$

The formula is normalized by simplifying the operation and ignoring those multiplication terms that are constant with respect to *y*, as is shown below:

(10) $$\begin{array}{c} {\tilde{p}}_{\text{ensemble}}\left(Y=y\;\left|\;v\right.\right)\propto \sqrt[{2^{n}}]{\underset{d\in {\left\{0,1\right\}}^{n}}{\prod }\exp\left(W_{y,:}^{T}\left(d\;⊙\;v\right)+b\right)},\\ =\exp\left(\frac{1}{2}\underset{d\in {\left\{0,1\right\}}^{n}}{\sum }W_{y,:}^{T}\left(d\;⊙\;v\right)+b\right),\\ =\exp\left(\frac{1}{2}W_{y,:}^{T}v+b\right). \end{array}$$

**Figure 4:**
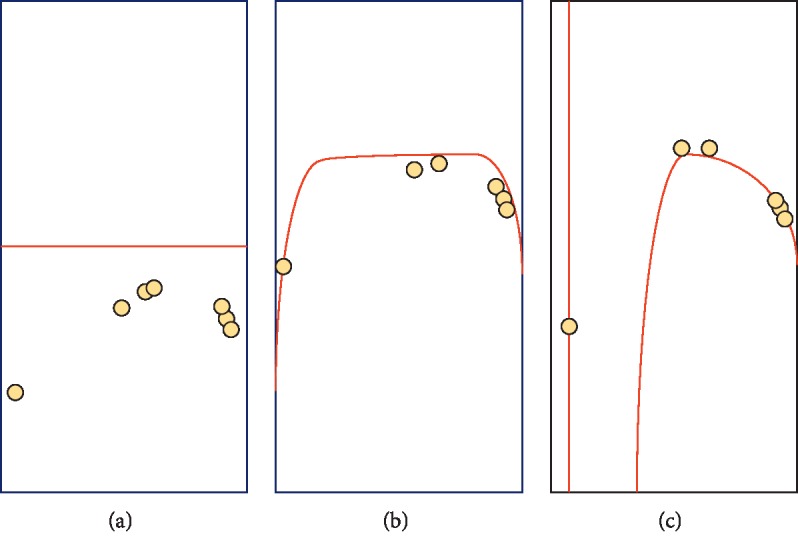
The trend of the regularizer. (a) Underfitting (large *λ*); (b) normal (proper *λ*); and (c) overfitting (small *λ*).

In order to reduce the error in classification, network parameters are regularized in the course of the training stage. The choice of regularization is to add weight delay to rectify the training standard of linear regression. The influence of regularization is shown in Figure 4.

L^2^-regularization is a good choice in the network. L^2^-regularization is also known as ridge regression or Tikhonov regularization and can be defined as the objective function as

(11) $$\begin{array}{c} \tilde{J}\left(\omega ,X,y\right)=\frac{\partial }{2}\omega^{T}\omega +J\left(\omega ,X,y\right), \end{array}$$

where *w* ^*T*^ *w* is the regularized term and *λ* is the value of weight decay. $\tilde{J}\left(\cdot \right)$ is the target function. We assume that there are no bias parameters with the corresponding parameter gradient.

(12) $$\begin{array}{c} \Omega \left(\theta \right)=\frac{1}{2}{\left\|w\right\|}_{2}^{2}. \end{array}$$

To take a single gradient step to update the weights, we perform this update:

(13) $$\begin{array}{c} \omega \;⟵\;\omega -\in \left(\partial \omega +\nabla_{\omega }J\left(\omega ;X,y\right)\right),\\ \text{or }\omega \;⟵\;\left(1-\;\in \;\partial \right)\omega -\in \nabla_{\omega }J\left(\omega ;X,y\right). \end{array}$$

This regularization strategy drives the weights closer to the origin by adding a regularization term *Ω*(*θ*)=(1/2)‖*w*‖_2_^2^ to the objective function. The addition of the weight decay term has modified the learning rule to multiplicatively shrink the weight vector by a constant factor on each step, just before performing the usual gradient update.

### 3.4. Dropout

Overfitting is a common problem in machine learning matters. In order to further solve the problem of overfitting, we usually adopt the integration that trains multiple models and combines their advantages together. The problem is that the model is time consuming to train and test. Dropout strategy can effectively alleviate the occurrence of overfitting and achieve regularization. Generally, dropout can be used as a trick for training deep networks. In this paper, dropout is adopted after the final MS-block network training. The contributions of dropout activation are as follows: the firstly is the averaging effect. The strategy can effectively prevent the problem of overfitting, and the random deletion of half of the hidden neurons leads to a different network structures, and the whole dropout process is equivalent to averaging a host of different neural networks. The second is reducing complex coadaptation relationships between neurons in the network. The updating of weights is no longer dependent on the coaction of implicit nodes with fixed relationships, which prevents some features from being effective only under other specific features, forcing the network to learn glowingly robust features, which also exist in random subsets of other neurons.

## 4. Transfer Learning

**Figure 5:**
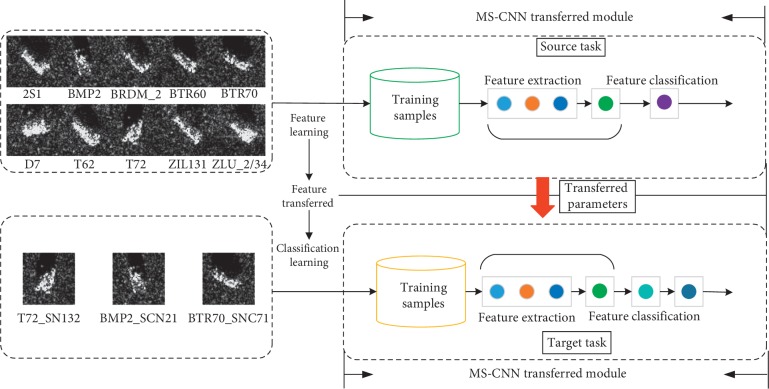
Transferred MS-CNN model.

Transfer learning is the ability of a system to recognize and apply knowledge and skills learned in previous domains/tasks to novel tasks/domains, which share some commonality. Given a source domain and source learning task, a target domain, and a target learning task, transfer learning aims to help improve the learning of the target predictive function *f* (*T*(·)) using the source knowledge, where *D*_*S*_ ≠ *D*_*T*_ or *T*_*S*_ ≠ *T*_*T*_. The domain consists of two components: a feature space *X* = {*x*_1_, *x*_2_,…, *x*_*n*_} and a marginal distribution *P* (*X*). Given a specific domain and label space *Y* = {*y*_1_,…, *y*_*n*_}, for each *x*_*i*_ in the domain, the task is to predict its corresponding label *y*_*i*_ where *y*_*i*_ ∈ *Y.* In general, if two tasks are different, then they may have different label spaces or different conditional distributions *P* (*Y* | *X*). Specifically, for SAR ATR task, the domain task shares the same feature space *F* = {*f*_*1*_,…, *f*_*n*_} with *n* dimensions, while the marginal probability distribution is different due to different classification task. In this paper, transfer learning configurations are implemented in Figure 5.

The algorithm of transfer learning is shown as follows: for the input, given the source dataset *D*_*S*_ and target dataset *D*_*T*_, set the initialized MS-CNN model *M*_Pre_ = *f* (*x*, *θ*_Pre_). Firstly, fine-tune the MS-CNN model *M*_0_ based on *M*_Pre_ using *D*_*S*_ to get a well-pretrained MS-CNN model. Then, transfer the shallow layer's parameters by freezing the learned layer, and the hyperparameters of MS-CNN are retrained on *D*_*T*_ until the model converges to the optimal solution. Specifically, for example, as transferred the parameters of conv4, the parameter update of the layer before conv4 would be the pretrained parameters, and the parameters of the rest layers would be trained from scratch. Finally, the small learning rate are considered to further fine tune the model slightly, to make the model more suitable for the SAR ATR task.

## 5. Experiments

All the experiments here are conducted on deep learning acceleration computing service with Intel Core i3-7350K CPU, on an Ubuntu 16.04 LTS operation system. The graphics card is NVIDIA GTX 1080ti, and the RAM is 8G. All the proposed convolutional neural network models are implemented using the publicly available Caffe framework.

### 5.1. Dataset

**Figure 6:**
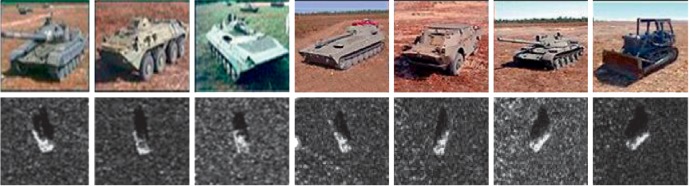
SAR image instance.

In this paper, experiments are conducted on the MSTAR dataset which was derived from the MSTAR project. Training and testing samples consisting of T72 (Main Battle Tanks), BMP2 (Armored Personal Carriers), and BTR70 (Armored Personal Carriers) are utilized here. The total number of samples was 698 images. The optical images and SAR images are shown in Figure 6. Dataset configuration is listed in Tables 2 and 3.

### 5.2. Training/Validation Methodology

**Figure 7:**
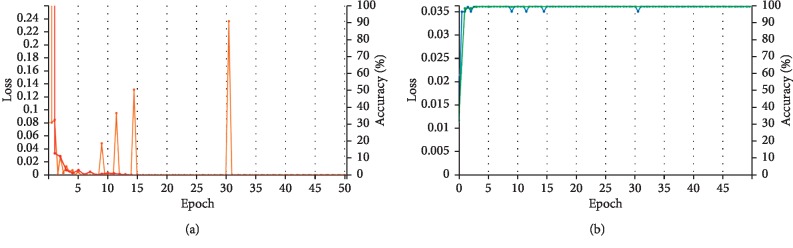
Predicted train/validation loss value. (a) Train/validation loss; (b) train/validation accuracy.

**Figure 8:**
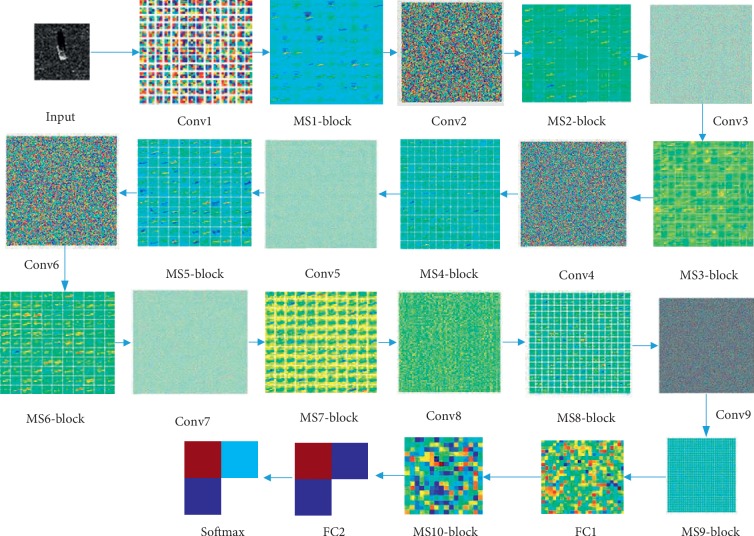
Visualization of the proposed network.

In the training process, 50 epochs are set to fine-tune the proposed models and information regarding about the loss values and output classification results is recorded. As shown in Figure 7, the accuracy of training and validation is 100%, and the predicted loss value converges to zero fast at an earlier time quantum. To evaluate the proposed model, we visualize the network layer by layer, which is shown in Figure 8. Observing the feature map from Conv1, the model learned some fundamental features from the input, such as edges and corners from all directions. The deeper the layers, the more complex and richer the learned features, including outlines, background, and higher-level semantic information. Features in the later layers are more discriminative containing the corresponding label information; thus, the learned feature in feature map is more targeted based on the belonging class.

### 5.3. Performance Evaluation

#### 5.3.1. Evaluation on Various MS Blocks

**Figure 9:**
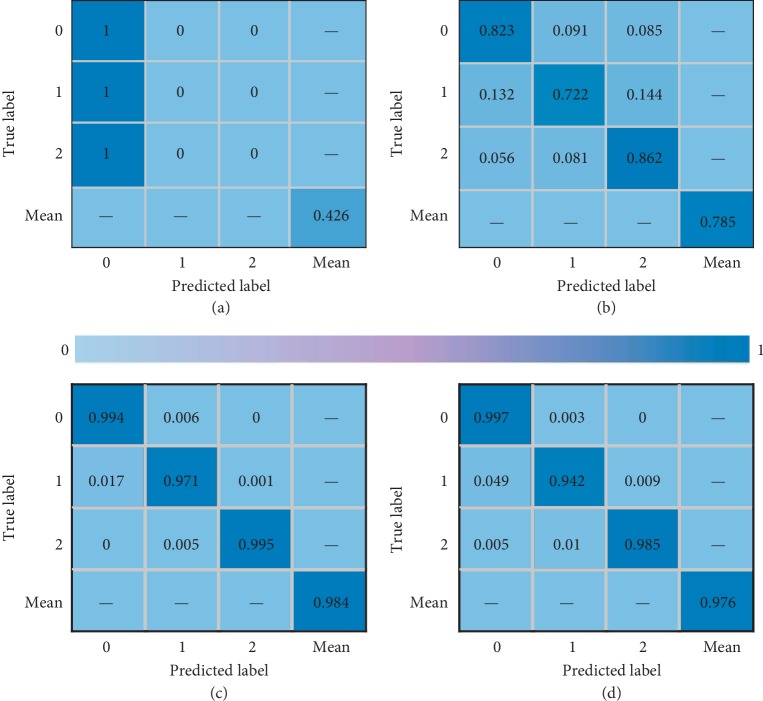
Confusion matrix-test dataset. (a) Six blocks; (b) eight blocks; (c) ten blocks; and (d) twelve blocks.

Performance on various blocks of MS-CNN is explored for the designed model. A different network model is reconstructed to validate on SAR image databases, and the accuracy of network feature extraction under variables is tested. The confusion matrix of the test dataset is shown in Figure 9. It is apparent that the network did not work efficiently as the Max-slice number is six, as the overfitting is caused by inadequate feature selection. From eight MS-blocks to twelve MS-blocks, the performance is relatively superior gradually and acquires excellent result at ten MS-blocks.

#### 5.3.2. Evaluation of Data Amplification

Ten MS-blocks are selected in the proposed network for further performance evaluation. We performed both the operations on data process and without pooling on the chosen network and noticed that the algorithm adopted quite excellently served to the model. As shown in Table 4, results without ROI extraction and data amplification are 74.51% and 80.66%, respectively.

#### 5.3.3. Evaluation on Various Activation Function

**Figure 10:**
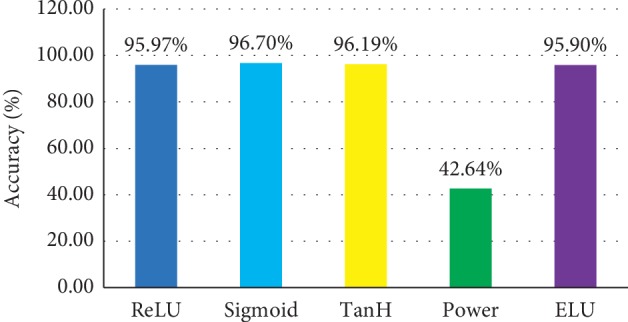
Model performance evaluation on various activation functions.

Meanwhile, motivated by the advantage that activation function contributes to advancing the understanding of the obtained features and boosting the performance of classification, ablation study on MS-CNN is also processed in this paper. The traditional activation function, such as ReLU, TanH, and Sigmoid, does not yield the top accuracy as expected. The Power function is negative due to the logistics operation that undermines the amount of the output features. The ELU is a common idea, and compared with the negative value of ReLU, the average value of cell activation ELU can be close to zero, similar to the effect of batch normalization but with lower computational complexity and soft saturation, and the accuracy of ELU is 95.93%. The model with sigmoid activation could achieve 96.70 accuracy. The result is shown in Figure 10.

#### 5.3.4. Evaluation on Dropout and Regularization

To get insight into the strategy, the comparable study was conducted as follows in Table 5. The accuracy with dropout strategy is 98.93%, boosting 1.64% compared with the one without dropout. As indicated in Table 6, L^2^-regularization has the advantages to enhance the performance by reducing the redundancy via adjusting the weighted delay. The L^1^-regularization method achieved 90.40% accuracy, reducing 8.53% improvement by using the L^2^-regularization. It has demonstrated that the L^2^-regularization has obtained excellent accuracy compared to other options, especially the method without any regularization that caused overfitting during the training stage.

### 5.4. Model Generalization and Robustness

#### 5.4.1. Generalization on Various Classes

Given the pretrained model which is optimized on the MS-CNN, it is supposed to acquire a good fit performance on the training samples, but also expected that our provided pretrained model shares good generalization ability on an unknown dataset. The intuitive manifestation of metrics measurement is the fitting performance and prediction performance on the unknown dataset. To this regard, we selected the optimized MS-CNN as the pretrained model to learn some sort of information from various classes SAR images classification. Specifically, we fine-tune the model by keeping all the layers before the last fc layer fixed and modifying the three classes as six, eight, and ten to attain more generic features.

Table 7 shows the generalization evaluation performance. The involved classes are randomly selected from the ten classes SAR images. From class three to class ten, the accuracy is all satisfactory, and they are 98.97%, 97.57%, 97.26%, and 94.19%, while the performance using the pretrained model surpass the one without it, and the improvements are 0.97%, 1.35%, and 2.59% from class six to class ten. Initiatively, the performance by using the pretrained MS-CNN model outperforms the one trained from scratch.

#### 5.4.2. Robustness Evaluation

**Figure 11:**
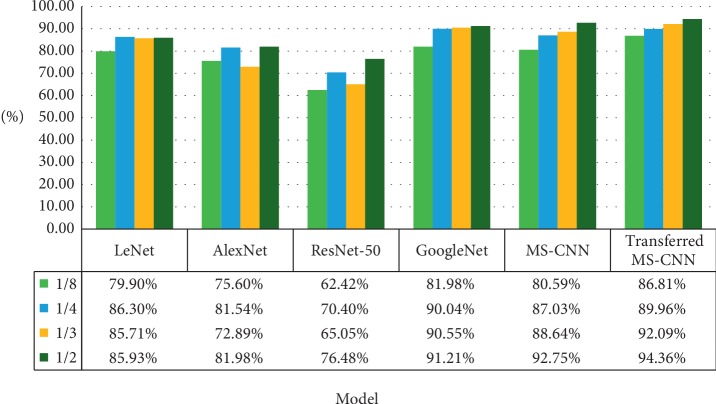
Result on state-of-the-art model on small samples.

*(1) Comparison to the State-of-the-Art Based on Small Samples*. Inspired by the superior performance on three target classification tasks, we carried on the experiments on different state-of-the-art networks. We randomly select 1/2, 1/3, 1/4, and 1/8 images from the raw training samples and enrich the dataset by the method proposed in Section 3. The aim is to validate the robustness of MS-CNN by reducing the raw images. Observed from Figure 11, the performance of our proposed method is better than the LeNet, AlexNet, and ResNet when applied to the SAR ATR. Generally, the deep structure is not satisfactory to deal with the simple images characterized with grayscale and small size, while our proposed method can achieve the corresponding result as the GoogleNet (VGG16).

*(2) Transfer Learning vs Scratch*. As is shown in Figure 11, from LeNet to MS-CNN, we notice that our proposed model could outperform partial networks, while some results are not satisfactory. Another brick in the wall is introducing the transfer learning approach. We trained the MS-CNN by ten class SAR images and transfered the weights to the three SAR image classification. From MS-CNN to MS-CNN (Transfer learning) in Figure 11, we notice that the model used transfer learning that could significantly surpass the performance of the model trained from scratch. Specifically, it has achieved an improvement over the current MS-CNN by 6.22%, 2.93%, 3.34%, and 1.61% in the 1/8, 1/4, 1/3, and 1/2 dataset. This observation validates the effectiveness of the transfer learning. The model trained from scratch is not satisfactory than the model using the pretrained model.

### 5.5. Performance Comparison with State-of-the-Art Algorithms

The performance comparison is illustrated in Table 8. The traditional method, such as SVM [41], has reached the accuracy of 90.00% in the SAR ATR. The performance of AE&LSVM [42] is the same as DNPP-L1 [43], about 94.14%. AF-CNN [44] is an additional feature-based CNN architecture which does not need additional preprocessing process or pose information, which boosts 4.38% improvement compared with SVM. The traditional CNN [46] could achieve 95.90% applied into SAR ATR, which is 0.77% lower than the unsupervised K-means & data amplification [47] method. SARNet [48] is a lightweight CNN model, which obtained the result of 98.30%. Performance on JLSND&SRC
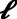
1 [24] is little inferior than that of our proposed method, about 98.89%. As shown in Table 8, compared with other state-of-the-art approaches, the proposed methods have obtained better recognition performance.

## 6. Conclusion

In this paper, a novel transferred MS-CNN structure with L^2^-regularization is proposed to solve the overfitting problem caused by the insufficient samples and model design for computational consumption. The data process pipeline is employed to address the data acquisition limitation and reduces the computation and redundancy for SAR target recognition. It is testified that combining the ROI extraction and data amplification algorithm has potential advantages to solve the sample problems. The transferred MS-CNN structure is available to refine the extracted features and contributes to SAR ATR. Furthermore, the methodology of dropout strategy and regularization term in this model has reflected despeciation to avoid the overfitting phenomenon. Overall, the performance conducted on extended MSTAR dataset indicates that our method is discriminative and effective and also proves that our proposed method is of good regularization and robustness. Due to the computational complexity and insufficient samples, a more efficient method, such as transfer leaning, a few shot learning will be explored in our future work.

## Figures and Tables

**Table 1:** Data visualization of the proposed MS-CNN.

Description	Data shape	Filter size/stride/pad	Depth	#Parameter
Input image	[1 64 64]	—	—	—
Conv1	[192 1 3 3]	3 × 3/1/1	1	1920
MS1-block	[96 64 64]	—	—	—
Conv2	[192 96 1 1]	1 × 1/1/0	1	18624
MS2-block	[96 32 32]	—	—	—
Conv3	[384 96 3 3]	3 × 3/1/1	1	332160
MS3-block	[192 32 32]	—	—	—
Conv4	[384 192 1 1]	1 × 1/1/0	1	74112
MS4-block	[192 16 16]	—	—	—
Conv5	[256 192 3 3]	3 × 3/1/1	1	442624
MS5-block	[128 16 16]	—	—	—
Conv6	[256 128 1 1]	1 × 1/1/0	1	33024
MS6-block	[128 16 16]	—	—	—
Conv7	[256 128 3 3]	3 × 3/1/1	1	295168
MS7-block	[128 16 16]	—	—	—
Conv8	[512 128 1 1]	1 × 1/1/0	1	66048
MS8-block	[256 8 8]	—	—	—
Conv9	[2048 256 1 1]	1 × 1/1/0	1	526336
MS9-block	[1024 8 8]	—	—	—
Fc1	[512 24576]	—	0	12583424
MS10-block	[256]	—	—	—
Fc2	[3 256]	—	0	771

**Table 2:** Configuration of MSTAR three-target database.

Training set	Number	Testing set	Number
T72_SN132	232	T72_SN132	196
		T72_SN812	195
		T72_SNS7	191
BMP2_SCN21	233	BMP2_SCN21	196
		BMP2_SCN9563	195
		BMP2_SCN9566	196
BTR70_SNC71	233	BTR70_SNC71	196

**Table 3:** Configuration of extended MSTAR three-target database.

Training set	Number	Testing set	Number
T72_SN132	84, 912	T72_SN132	196
		T72_SN812	195
		T72_SNS7	191
BMP2_SCN21	85, 278	BMP2_SCN21	196
		BMP2_SCN9563	195
		BMP2_SCN9566	196
BTR70_SNC71	85, 278	BTR70_SNC71	196

**Table 4:** Model performance on ROI extraction and data amplification variation.

Model	ROI	Data amplification	Accuracy (%)
MS-CNN	No	No	63.30
	No	Yes	74.51
	Yes	No	80.60
	Yes	Yes	98.93

**Table 5:** Modelling performance of dropout strategy.

Model	Dropout	Accuracy (%)
MS-CNN	No	97.29
	Yes	98.93

**Table 6:** Modelling performance of various regularizations.

Model	L^1^-regularization	L^2^-regularization	Accuracy (%)
MS-CNN	No	No	Overfitting
	Yes	No	90.40
	No	Yes	98.93

**Table 7:** Generalization on various classes.

Class number	Pretrained model	Accuracy (%)
3	Without	98.97
	With	—
6	Without	97.57
	With	98.54
8	Without	97.26
	With	98.61
10	Without	94.19
	With	96.78

**Table 8:** Performance comparison with other proposed methods.

Method	Accuracy (%)
SVM [41]	90.00
AlexNet	93.55
AE&LSVM [42]	94.14
DNPP-L1 [43]	94.14
AF-CNN [44]	94.38
Gabor & LPQ & ELM [45]	94.80
CNN [46]	95.90
Unsupervised K-means & data amplification [47]	96.67
LeNet [48]	97.29
ResNet-50 [24]	97.66
SARNet [49]	98.30
JLSND&SRC 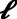 _1_ [50]	98.30
Proposed	98.93

## Data Availability

The data used to support the findings of this study are available from the corresponding author upon request.
